# Nonclassicality by Local Gaussian Unitary Operations for Gaussian States

**DOI:** 10.3390/e20040266

**Published:** 2018-04-11

**Authors:** Yangyang Wang, Xiaofei Qi, Jinchuan Hou

**Affiliations:** 1 Department of Mathematics, Shanxi University, Taiyuan 030006, China; 2 Institute of Big Data Science and Industry, Shanxi University, Taiyuan 030006, China; 3 Department of Mathematics, Taiyuan University of Technology, Taiyuan 030024, China

**Keywords:** quantum correlations, Gaussian states, Gaussian unitary operations, continuous-variable systems

## Abstract

A measure of nonclassicality N in terms of local Gaussian unitary operations for bipartite Gaussian states is introduced. N is a faithful quantum correlation measure for Gaussian states as product states have no such correlation and every non product Gaussian state contains it. For any bipartite Gaussian state $\rho_{AB}$, we always have $0\leq N(\rho_{AB})<1$, where the upper bound 1 is sharp. An explicit formula of N for (1+1)-mode Gaussian states and an estimate of N for (n+m)-mode Gaussian states are presented. A criterion of entanglement is established in terms of this correlation. The quantum correlation N is also compared with entanglement, Gaussian discord and Gaussian geometric discord.

## 1. Introduction

The presence of correlations in bipartite quantum systems is one of the main features of quantum mechanics. The most important one among such correlations is entanglement [1]. However, recently much attention has been devoted to the study and the characterization of quantum correlations that go beyond the paradigm of entanglement, being necessary but not sufficient for its presence. Non-entangled quantum correlations also play important roles in various quantum communications and quantum computing tasks [2,3,4,5].

For the last two decades, various methods have been proposed to quantify quantum correlations, such as quantum discord (QD) [6,7], geometric quantum discord [8,9], measurement-induced nonlocality (MIN) [10] and measurement-induced disturbance (MID) [11] for discrete-variable systems. It is also important to develop new simple criteria for witnessing correlations beyond entanglement for continuous-variable systems. In this direction, Giorda, Paris [12] and Adesso, Datta [13] independently introduced the definition of Gaussian QD for Gaussian states and discussed its properties. Adesso and Girolami in [14] proposed the concept of Gaussian geometric discord (GD) for Gaussian states. Measurement-induced disturbance of Gaussian states was studied in [15], while MIN for Gaussian states was discussed in [16]. For other related results, see [17,18] and the references therein. Note that not every quantum correlation defined for discrete-variable systems has a Gaussian analogy for continuous-variable systems [16]. On the other hand, the values of Gaussian QD and Gaussian GD are very difficult to be computed and the known formulas are only for some (1+1)-mode Gaussian states. Little information is revealed by Gaussian QD and GD. The purpose of this paper is to introduce a new measure of nonclassicality for (n+m)-mode quantum states in continuous-variable systems, which is simpler to be computed and can be used with any (n+m)-mode Gaussian states.

Given a bipartite quantum state ρ acting on Hilbert space $H_{A}⊗H_{B}$, denote by $\rho_{A}={\mathrm{Tr}}_{B}\left(\rho \right)$ the reduced density operator in subsystem A. For the case of finite dimensional systems, the author of [19] proposed a quantity $d_{U_{A}}\left(\rho \right)$ defined by $d_{U_{A}}\left(\rho \right)=\frac{1}{\sqrt{2}}{∥\rho -\left(U_{A}⊗I\right)\rho {\left(U_{A}⊗I\right)}^{†}∥}_{F},$ where ${∥A∥}_{F}=\sqrt{\mathrm{Tr}(A^{†}A)}$ denotes the Frobenius norm and $U_{A}$ is any unitary operator satisfying $[\rho_{A},U_{A}]=0$. This quantity demands that the reduced density matrix of the subsystem A is invariant under this unitary transformation. However, the global density matrix may be changed after such local unitary operation, and therefore $d_{U_{A}}\left(\rho \right)$ may be non-zero for some $U_{A}$. Then, Datta, Gharibian, et al. discussed respectively in [20,21] the properties of $d_{U_{A}}\left(\rho \right)$ and revealed that $\max_{U_{A}}d_{U_{A}}\left(\rho \right)$ can be used to investigate the nonclassical effect.

Motivated by the works in [19,20,21], we can consider an analogy for continuous-varable systems. In the present paper, we introduce a quantity N in terms of local Gaussian unitary operations for (n+m)-mode quantum states in Gaussian systems. Different from the finite dimensional case, besides the local Gaussian unitary invariance property for quantum states, we also show that $N(\rho_{AB})=0$ if and only if $\rho_{AB}$ is a Gaussian product state. This reveals that the quantity N is a kind of faithful measure of the nonclassicality for Gaussian states that a state has this nonclassicality if and only if it is not a product state. In addition, we show that $0\leq N(\rho_{AB})<1$ for each (n+m)-mode Gaussian state $\rho_{AB}$ and the upper bound 1 is sharp. An estimate of N for any (n+m)-mode Gaussian states is provided and an explicit formula of N for any (1+1)-mode Gaussian states is obtained. As an application, a criterion of entanglement for (1+1)-mode Gaussian states is established in terms of N by numerical approaches. Finally, we compare N with Gaussian QD and Gaussian GD to illustrate that it is a better measure of the nonclassicality.

## 2. Gaussian States and Gaussian Unitary Operations

Recall that, for arbitrary state ρ in an *n*-mode continuous-variable system, its characteristic function $\chi_{\rho }$ is defined as

$$\chi_{\rho }\left(z\right)=\mathrm{Tr}\left(\rho W\left(z\right)\right),$$

where $z={\left(x_{1},y_{1},\cdots ,x_{n},y_{n}\right)}^{T}\in R^{2n}$ with R the field of real numbers and ${\left(\cdot \right)}^{T}$ the transposition, and $W\left(z\right)=\exp\left(iR^{T}z\right)$ is the Weyl operator. Let $R={\left(R_{1},R_{2},\cdots ,R_{2n}\right)}^{T}={\left({\hat{Q}}_{1},{\hat{P}}_{1},\cdots ,{\hat{Q}}_{n},{\hat{P}}_{n}\right)}^{T}$. As usual, ${\hat{Q}}_{i}$ and ${\hat{P}}_{i}$ stand respectively for the position and momentum operators for each i∈{1,2,⋯,n}. They satisfy the Canonical Commutation Relation (CCR) in natural units (ℏ=1)

$$\left[{\hat{Q}}_{i},{\hat{P}}_{j}\right]=\delta_{ij}iI\;\;\mathrm{and}\;\;\left[{\hat{Q}}_{i},{\hat{Q}}_{j}\right]=\left[{\hat{P}}_{i},{\hat{P}}_{j}\right]=0,$$

i,j=1,2,…,n.

*Gaussian states:* ρ is called a Gaussian state if $\chi_{\rho }\left(z\right)$ is of the form

$$\begin{array}{c} \chi_{\rho }\left(z\right)=\exp\left[-\frac{1}{4}z^{T}\Gamma z+id^{T}z\right], \end{array}$$

where

$$\begin{array}{cc} d= & {\left(\langle {\hat{R}}_{1}\rangle ,\langle {\hat{R}}_{2}\rangle ,\ldots ,\langle {\hat{R}}_{2n}\rangle \right)}^{T}\\ = & {\left(\mathrm{Tr}\left(\rho R_{1}\right),\mathrm{Tr}\left(\rho R_{2}\right),\ldots ,\mathrm{Tr}\left(\rho R_{2n}\right)\right)}^{T}\in R^{2n} \end{array}$$

is called the mean or the displacement vector of ρ and $\Gamma =\left(\gamma_{kl}\right)\in M_{2n}\left(R\right)$ is the covariance matrix (CM) of ρ defined by $\gamma_{kl}=\mathrm{Tr}\left[\rho \left(\Delta {\hat{R}}_{k}\Delta {\hat{R}}_{l}+\Delta {\hat{R}}_{l}\Delta {\hat{R}}_{k}\right)\right]$ with $\Delta {\hat{R}}_{k}={\hat{R}}_{k}-\langle {\hat{R}}_{k}\rangle$ ([22,23,24]). Here, $M_{l\times k}\left(R\right)$ stands for the set of all *l*-by-*k* real matrices and, when l=k, we write $M_{l\times k}\left(R\right)$ as $M_{l}\left(R\right)$. Note that the CM Γ of a state is symmetric and must satisfy the uncertainty principle Γ+iΔ≥0, where $\Delta ={⊕}_{i=1}^{n}\Delta_{i}$ with $\Delta_{i}=\left(\begin{array}{cc} 0 & 1\\ -1 & 0 \end{array}\right)$ for each *i*. From the diagonal terms of the above inequality, one can easily derive the usual Heisenberg uncertainty relation for position and momentum $V\left({\hat{Q}}_{i}\right)V\left({\hat{P}}_{i}\right)\geq 1$ with $V\left(\hat{R_{i}}\right)=\langle {\left(\Delta \hat{R_{i}}\right)}^{2}\rangle$ [25].

Now assume that $\rho_{AB}$ is any (n+m)-mode Gaussian state. Then, the CM Γ of $\rho_{AB}$ can be written as

(1) $$\begin{array}{c} \Gamma =\left(\begin{array}{cc} A & C\\ C^{T} & B \end{array}\right), \end{array}$$

where $A\in M_{2n}\left(R\right)$, $B\in M_{2m}\left(R\right)$ and $C\in M_{2n\times 2m}\left(R\right)$. Particularly, if n=m=1 , by means of local Gaussian unitary (symplectic at the CM level) operations, Γ has a standard form:

(2) $$\begin{array}{c} \Gamma_{0}=\left(\begin{array}{cc} A_{0} & C_{0}\\ C_{0}^{T} & B_{0} \end{array}\right), \end{array}$$

where $A_{0}=\left(\begin{array}{cc} a & 0\\ 0 & a \end{array}\right)$, $B_{0}=\left(\begin{array}{cc} b & 0\\ 0 & b \end{array}\right)$, $C_{0}=\left(\begin{array}{cc} c & 0\\ 0 & d \end{array}\right)$, $\Gamma_{0}>0$, $\det\Gamma_{0}\geq 1$ and $\det\Gamma_{0}+1\geq \det A_{0}+\det B_{0}+2\det C_{0}$ ([26,27,28,29]).

*Gaussian unitary operations.* Let us consider an *n*-mode continuous-variable system with $R={\left({\hat{Q}}_{1},{\hat{P}}_{1},\cdots ,{\hat{Q}}_{n},{\hat{P}}_{n}\right)}^{T}$. For a unitary operator *U*, the unitary operation $\rho \mapsto U\rho U^{†}$ is said to be Gaussian if its output is a Gaussian state whenever its input is a Gaussian state, and such *U* is called a Gaussian unitary operator. It is known that a unitary operator *U* is Gaussian if and only if

$$\begin{array}{c} U^{†}RU=SR+m, \end{array}$$

for some vector m in $R^{2n}$ and some S∈Sp(2n,R), the symplectic group of all 2n×2n real matrices S that satisfy

$$S\in \mathrm{Sp}\left(2n,R\right)\Leftrightarrow S\Delta S^{T}=\Delta .$$

Thus, every Gaussian unitary operator *U* is determined by some affine symplectic map (S,m) acting on the phase space, and can be denoted by $U=U_{S,m}$ ([23,24]).

The following well-known facts for Gaussian states and Gaussian unitary operations are useful for our purpose.

**Lemma** **1** ([23])**.** *For any (n+m)-mode Gaussian state $\rho_{AB}$, write its CM* Γ *as in Equation (1). Then, the CMs of the reduced states $\rho_{A}={\mathrm{Tr}}_{B}\rho_{AB}$ and $\rho_{B}={\mathrm{Tr}}_{A}\rho_{AB}$ are matrices A and B, respectively.*

Denote by $S(H_{A}⊗H_{B})$ the set of all quantum states of $H_{A}⊗H_{B}$, where $H_{A}$ and $H_{B}$ are respectively the state space for *n*-mode and *m*-mode continuous-variable systems.

**Lemma** **2** ([30])**.** *If $\rho_{AB}\in S\left(H_{A}⊗H_{B}\right)$ is an (n+m)-mode Gaussian state, then $\rho_{AB}$ is a product state, that is, $\rho_{AB}=\sigma_{A}⊗\sigma_{B}$ for some $\sigma_{A}\in S\left(H_{A}\right)$ and $\sigma_{B}\in S\left(H_{B}\right)$, if and only if $\Gamma =\Gamma_{A}⊕\Gamma_{B}$, where* Γ*, $\Gamma_{A}$ and $\Gamma_{B}$ are the CMs of $\rho_{AB}$, $\sigma_{A}$ and $\sigma_{B}$, respectively.*

**Lemma** **3** ([23,24])**.** *Assume that ρ is any n-mode Gaussian state with CM* Γ *and displacement vector d, and $U_{S,m}$ is a Gaussian unitary operator. Then, the characteristic function of the Gaussian state $\sigma =U\rho U^{†}$ is of the form $\exp(-\frac{1}{4}z^{T}\Gamma_{\sigma }z+id_{\sigma }^{T}z)$, where $\Gamma_{\sigma }=S\Gamma S^{T}$ and $d_{\sigma }=m+Sd$.*

## 3. Quantum Correlation Introduced by Gaussian Unitary Operations

Now, we introduce a quantum correlation N by local Gaussian unitary operations in the continuous-variable system.

**Definition** **1.** *For any (n+m)-mode quantum state $\rho_{AB}\in S\left(H_{A}⊗H_{B}\right)$, the quantum correlation $N(\rho_{AB})$ of $\rho_{AB}$ by Gaussian unitary operations is defined by*

(3) $$\begin{array}{c} N\left(\rho_{AB}\right)=\frac{1}{2}\underset{U}{\sup}{∥\rho_{AB}-\left(I⊗U\right)\rho_{AB}\left(I⊗U^{†}\right)∥}_{2}^{2}, \end{array}$$

*where the supremum is taken over all Gaussian unitary operators $U\in B(H_{B})$ satisfying $U\rho_{B}U^{†}=\rho_{B}$, and $\rho_{B}={\mathrm{Tr}}_{A}\left(\rho_{AB}\right)$ is the reduced state. Here, $B(H_{B})$ is the set of all bounded linear operators acting on $H_{B}$.*

Observe that $N(\rho_{AB})=0$ holds for every product state. Thus, the product state contains no such correlation.

**Remark** **1.** For any Gaussian state $\rho_{AB}$, there exist many Gaussian unitary U so that $U\rho_{B}U^{†}=\rho_{B}$. This ensures that the definition of the quantity $N(\rho_{AB})$ makes sense for each Gaussian state $\rho_{AB}$.

To see this, we need Williamson Theorem ([31]), which states that, for any *n*-mode Gaussian state ρ∈S(H) with CM $\Gamma_{\rho }$, there exists a 2n×2n symplectic matrix S such that $S\Gamma_{\rho }S^{T}={⊕}_{i=1}^{n}v_{i}I_{2}$ with $v_{i}\geq 1$. The diagonal matrix ${⊕}_{i=1}^{n}v_{i}I_{2}$ and $v_{i}$s are called respectively the Williamson form and the symplectic eigenvalues of $\Gamma_{\rho }$. By the Williamson Theorem, there exists a Gaussian unitary operator $U=U_{S,m}=U_{S,-Sd}$ such that $U\rho U^{†}={⊗}_{i=1}^{n}\rho_{i}$, where $\rho_{i}$ are thermal states. Let $S_{\theta }={⊕}_{i=1}^{n}S_{\theta_{i}}$ with $S_{\theta_{i}}=\left(\begin{array}{cc} \cos\theta_{i} & \sin\theta_{i}\\ -\sin\theta_{i} & \cos\theta_{i} \end{array}\right),$ $\theta_{i}\in \left[0,\frac{\pi }{2}\right]$. Then, $S_{\theta }$ is a symplectic matrix, and the corresponding Gaussian unitary operator $U_{S_{\theta },0}=U_{S_{\theta }}$ has the form $U_{S_{\theta }}={⊗}_{i=1}^{n}U_{S_{\theta_{i}}}={⊗}_{i=1}^{n}\exp\left(\theta_{i}{\hat{a}}_{i}^{†}{\hat{a}}_{i}\right)$. It is easily checked that $S_{\theta }\left({⊕}_{i=1}^{n}v_{i}I\right)S_{\theta }^{T}={⊕}_{i=1}^{n}v_{i}I$, and so $U_{S_{\theta }}\left({⊗}_{i=1}^{n}\rho_{i}\right)U_{S_{\theta }}^{†}={⊗}_{i=1}^{n}\rho_{i}$. Now, write $W=U^{†}U_{S_{\theta }}U$. Obviously, *W* is Gaussian unitary and satisfies $W\rho W^{†}=U^{†}U_{S_{\theta }}U\rho U^{†}U_{S_{\theta }}^{†}U=\rho$.

We first prove that N is local Gaussian unitary invariant for all quantum states.

**Proposition** **1** (Local Gaussian unitary invariance)**.** If $\rho_{AB}\in S\left(H_{A}⊗H_{B}\right)$ is an (n+m)-mode quantum state, then $N\left(\left(U⊗V\right)\rho_{AB}\left(U^{†}⊗V^{†}\right)\right)=N\left(\rho_{AB}\right)$ holds for any Gaussian unitary operators $U\in B(H_{A})$ and $V\in B(H_{B})$.

**Proof** **of** **Proposition** **1.** Let $\rho_{AB}\in S\left(H_{A}⊗H_{B}\right)$ be an (n+m)-mode Gaussian state. For any Gaussian unitary operators $U\in B(H_{A})$ and $V\in B(H_{B})$, denote $\sigma_{AB}=\left(U⊗V\right)\rho_{AB}\left(U^{†}⊗V^{†}\right)$. Then, $\sigma_{B}=V\rho_{B}V^{†}$. For any Gaussian unitary operator $W\in B(H_{B})$ satisfying $W\sigma_{B}W^{†}=\sigma_{B}$, we have $WV\rho_{B}V^{†}W^{†}=V\rho_{B}V^{†}$. Let $W^{\prime }=V^{†}WV$. Then, $W^{\prime }$ is also a Gaussian unitary operator and satisfies $W^{\prime }\rho_{B}W^{\prime †}=V^{†}WV\rho_{B}V^{†}W^{†}V=\rho_{B}$. It is clear that $W^{\prime }$ runs over all Gaussian unitary operators that commutes with $\rho_{B}$ when *W* runs over all Gaussian unitary operators commuting with $\sigma_{B}$. Hence, by Equation (3), we have

$$\begin{array}{cc} & N(\sigma_{AB})\\ = & \frac{1}{2}\underset{W}{\sup}{∥\sigma_{AB}-\left(I⊗W\right)\sigma_{AB}\left(I⊗W\right)∥}_{2}^{2}\\ = & \frac{1}{2}\underset{W}{\sup}{∥\left(U⊗V\right)\rho_{AB}\left(U^{†}⊗V^{†}\right)-\left(I⊗W\right)\left(U⊗V\right)\rho_{AB}\left(U^{†}⊗V^{†}\right)\left(I⊗W\right)∥}_{2}^{2}\\ = & \underset{W}{\sup}\left\{\mathrm{Tr}\left(\rho_{AB}^{2}\right)-\mathrm{Tr}\left(\rho_{AB}\left(I⊗V^{†}WV\right)\rho_{AB}\left(I⊗V^{†}W^{†}V\right)\right)\right\}\\ = & \underset{W^{\prime }}{\sup}\left\{\mathrm{Tr}\left(\rho_{AB}^{2}\right)-\mathrm{Tr}\left(\rho_{AB}\left(I⊗W^{\prime }\right)\rho_{AB}\left(I⊗W^{\prime †}\right)\right)\right\}\\ = & \frac{1}{2}\underset{W^{\prime }}{\sup}{∥\rho_{AB}-\left(I⊗W^{\prime }\right)\rho_{AB}\left(I⊗W^{\prime †}\right)∥}_{2}^{2}\\ = & N(\rho_{AB}) \end{array}$$

as desired. ☐

The next theorem shows that $N(\rho_{AB})$ is a faithful nonclassicality measure for Gaussian states.

**Theorem** **1.** For any (n+m)-mode Gaussian state $\rho_{AB}\in S\left(H_{A}⊗H_{B}\right)$, $N(\rho_{AB})=0$ if and only if $\rho_{AB}$ is a product state.

**Proof** **of** **Theorem** **1.** By Definition 1, the “if” part is apparent. Let us check the “only if” part. Since the mean of any Gaussian state can be transformed to zero under some local Gaussian unitary operation, it is sufficient to consider those Gaussian states whose means are zero by Proposition 1. In the sequel, assume that $\rho_{AB}$ is an (n+m)-mode Gaussian state with zero mean vector and CM $\Gamma =\left(\begin{array}{cc} A & C\\ C^{T} & B \end{array}\right)$ as in Equation (1), so that $N(\rho_{AB})=0$.

By Lemma 1, the CM of $\rho_{B}$ is *B*. According to the Williamson Theorem, there exists a symplectic matrix $S_{0}$ such that $S_{0}BS_{0}^{T}={⊕}_{i=1}^{m}v_{i}I$ and $U_{0}\rho_{B}U_{0}^{†}={⊗}_{i=1}^{m}\rho_{i}$, where $U_{0}=U_{S_{0},0}$ and $\rho_{i}$ are of the thermal states. Write $\sigma_{AB}=\left(I⊗U_{0}\right)\rho_{AB}\left(I⊗U_{0}^{†}\right)$. It follows from Proposition 1 that $N\left(\sigma_{AB}\right)=N\left(\rho_{AB}\right)=0$. Obviously, $\sigma_{AB}$ has the CM of form:

$$\Gamma^{\prime }=\left(\begin{array}{cc} A^{\prime } & C^{\prime }\\ C^{\prime T} & {⊕}_{i}^{m}v_{i}I \end{array}\right)$$

and the mean 0.

For any $\theta_{i}\in \left[0,\frac{\pi }{2}\right]$ for i=1,2,⋯,m, let $S_{\theta }$ be the symplectic matrix as in Remark 1. Then, $S_{\theta }\left({⊕}_{i=1}^{m}v_{i}I\right)S_{\theta }^{T}={⊕}_{i=1}^{m}v_{i}I$ and $U_{S_{\theta },0}\sigma_{B}U_{S_{\theta },0}^{†}=\sigma_{B}={\mathrm{Tr}}_{A}\left(\sigma_{AB}\right)$. As $N(\sigma_{AB})=0$, by Equation (3), $\sigma_{AB}=\left(I⊗U_{S_{\theta },0}\right)\sigma_{AB}\left(I⊗U_{S_{\theta },0}^{†}\right)$, and hence they must have the same CMs, that is,

$$\begin{array}{c} \left(\begin{array}{cc} A^{\prime } & C^{\prime }\\ C^{\prime T} & {⊕}_{i=1}^{m}v_{i}I \end{array}\right)=\left(\begin{array}{cc} A^{\prime } & C^{\prime }S_{\theta }^{T}\\ S_{\theta }C^{\prime T} & {⊕}_{i=1}^{m}v_{i}I \end{array}\right). \end{array}$$

Note that $I-S_{\theta }^{T}$ is an invertible matrix if we take $\theta_{i}\in \left(0,\frac{\pi }{2}\right)$ for each *i*. Then, it follows from $C^{\prime }=C^{\prime }S_{\theta }^{T}$ that we must have $C^{\prime }=0.$ Thus, $\sigma_{AB}$ is a product state by Lemma 2, and, consequently, $\rho_{AB}=\left(I⊗U_{0}^{†}\right)\sigma_{AB}\left(I⊗U_{0}\right)$ is also a product state. ☐

We can give an analytic formula of $N(\rho_{AB})$ for (1+1)-mode Gaussian state $\rho_{AB}$. Since N is locally Gaussian unitary invariant, it is enough to assume that the mean vector of $\rho_{AB}$ is zero and the CM is standard.

**Theorem** **2.** *For any (1+1)-mode Gaussian state $\rho_{AB}$ with CM *Γ* whose standard form is $\Gamma_{0}=\left(\begin{array}{cc} A_{0} & C_{0}\\ C_{0}^{T} & B_{0} \end{array}\right)$ as in Equation (2), we have*

(4) $$\begin{array}{c} N\left(\rho_{AB}\right)=\frac{1}{\sqrt{\left(ab-c^{2}\right)\left(ab-d^{2}\right)}}-\frac{1}{\sqrt{\left(ab-\frac{c^{2}}{2}\right)\left(ab-\frac{d^{2}}{2}\right)}}. \end{array}$$

*Particularly, $N\left(\rho_{AB}\right)=1-\sqrt{\frac{2}{2-c^{2}d^{2}+ab\left(c^{2}+d^{2}\right)}}$ whenever $\rho_{AB}$ is pure.*

**Proof** **of** **Theorem** **2.** By Proposition 1, we may assume that the mean vector of $\rho_{AB}$ is zero. Let $U_{S,m}$ be a Gaussian unitary operator such that $U_{S,m}\rho_{B}U_{S,m}^{†}=\rho_{B}$. Then, S and m meet the conditions $SB_{0}S^{T}=B_{0}$ and $Sd_{B}+m=d_{B}=0$. It follows that m=0. Thus, we can denote $U_{S,m}$ by $U_{S}$. As $S\Delta S^{T}=\Delta$, there exists some $\theta \in [0,\frac{\pi }{2}]$ such that $S=S_{\theta }=\left(\begin{array}{cc} \cos\theta & \sin\theta \\ -\sin\theta & \cos\theta \end{array}\right)$. Thus, the CM of Gaussian state $\left(I⊗U_{S}\right)\rho_{AB}\left(I⊗U_{S}^{†}\right)$ is

$$\begin{array}{c} \Gamma_{\theta }=\left(\begin{array}{cccc} a & 0 & c\cos\theta & -c\sin\theta \\ 0 & a & d\sin\theta & d\cos\theta \\ c\cos\theta & d\sin\theta & b & 0\\ -c\sin\theta & d\cos\theta & 0 & b \end{array}\right), \end{array}$$

and the mean of $\left(I⊗U_{S}\right)\rho_{AB}\left(I⊗U_{S}^{†}\right)$ is (I⊕S)d+0⊕0=0 as d=0. Hence, by Equations (3) and (4), one gets

$$\begin{array}{cc} & N(\rho_{AB})\\ = & \frac{1}{2}\underset{U_{S,m}}{\sup}{∥\rho_{AB}-\left(I⊗U\right)\rho_{AB}\left(I⊗U_{S,m}^{†}\right)∥}_{2}^{2}\\ = & \underset{U_{S,m}}{\sup}\left\{\mathrm{Tr}\left(\rho_{AB}^{2}\right)-\mathrm{Tr}\left(\rho_{AB}\left(I⊗U_{S,m}\right)\rho_{AB}\left(I⊗U_{S,m}^{†}\right)\right)\right\}\\ = & \underset{\theta \in [0,\frac{\pi }{2}]}{\sup}\left\{\frac{1}{\sqrt{\det\Gamma }}-\frac{1}{\sqrt{\det[\left(\Gamma +\Gamma_{\theta }\right)/2]}}\right\}\\ = & \max_{\theta \in [0,\frac{\pi }{2}]}\{\frac{1}{\sqrt{a^{2}b^{2}+c^{2}d^{2}-ab\left(c^{2}+d^{2}\right)}}\\ & -\frac{1}{\sqrt{\left[ab-c^{2}\left(1+\cos\theta \right)/2\right]\left[ab-d^{2}\left(1+\cos\theta \right)/2\right]}}\}\\ = & \frac{1}{\sqrt{\left(ab-c^{2}\right)\left(ab-d^{2}\right)}}-\frac{1}{\sqrt{\left(ab-c^{2}/2\right)\left(ab-d^{2}/2\right)}}. \end{array}$$

Hence, Equation (4) is true.

Particularly, if $\rho_{AB}$ is a pure state, then, by [29], we have $1=\mathrm{Tr}\left(\rho^{2}\right)=\frac{1}{\sqrt{det\Gamma }}=\frac{1}{\sqrt{\left(ab-c^{2}\right)\left(ab-d^{2}\right)}}.$ This entails that $N\left(\rho_{AB}\right)=1-\sqrt{\frac{2}{2-c^{2}d^{2}+ab\left(c^{2}+d^{2}\right)}}$. ☐

For the general (n+m)-mode case, it is difficult to give an analytic formula of $N(\rho_{AB})$ for all (n+m)-mode Gaussian states $\rho_{AB}$. However, we are able to give an estimate of $N(\rho_{AB})$.

**Theorem** **3.** *For any (n+m)-mode Gaussian state $\rho_{AB}$ with CM $\Gamma =\left(\begin{array}{cc} A & C\\ C^{T} & B \end{array}\right)$ as in Equation (1), we have*

(5) $$\begin{array}{c} 0\leq N\left(\rho_{AB}\right)\leq \frac{1}{\sqrt{\det\Gamma }}-\frac{1}{\sqrt{\left(\det A)(\det B\right)}}<1. \end{array}$$

*Particularly, when $\rho_{AB}$ is pure, $N\left(\rho_{AB}\right)\leq 1-\frac{1}{\sqrt{\left(\det A)(\det B\right)}}.$ Moreover, the upper bound 1 in the inequality (5) is sharp, that is, we have*

$$\underset{\rho_{AB}}{\sup}N\left(\rho_{AB}\right)=1.$$

**Proof** **of** **Theorem** **3.** By Proposition 1, without loss of generality, we may assume that the mean of $\rho_{AB}$ is 0. Let $U_{S,m}$ be a Gaussian unitary operator such that $U_{S,m}\rho_{B}U_{S,m}^{†}=\rho_{B}$. Then, the CM and the mean of the Gaussian state $\left(I⊗U_{S,m}\right)\rho_{AB}\left(I⊗U_{S,m}^{†}\right)$ are $\Gamma_{U}=\left(\begin{array}{cc} A & CS^{T}\\ SC^{T} & B \end{array}\right)$ and 0, respectively. Note that, for any *n*-mode Gaussian states ρ,σ with CMs $V_{\rho },V_{\sigma }$ and means $d_{\rho },d_{\sigma }$, respectively, it is shown in [32] that

(6) $$\begin{array}{c} \mathrm{Tr}\left(\rho \sigma \right)=\frac{1}{\sqrt{\det[\left(V_{\rho }+V_{\sigma }\right)/2]}}\exp\left[-\frac{1}{2}\delta {\langle d\rangle }^{T}\det{\left[\left(V_{\rho }+V_{\sigma }\right)/2\right]}^{-1}\delta \langle d\rangle \right],\;\mathrm{where}\;\delta \langle d\rangle =d_{\rho }-d_{\sigma }. \end{array}$$

Hence,

$$\begin{array}{cc} N(\rho_{AB})= & \frac{1}{2}\underset{U}{\sup}{∥\rho_{AB}-\left(I⊗U\right)\rho_{AB}\left(I⊗U^{†}\right)∥}_{2}^{2}\\ = & \underset{U}{\sup}\left\{\mathrm{Tr}\left(\rho_{AB}^{2}\right)-\mathrm{Tr}\left(\rho_{AB}\left(I⊗U\right)\rho_{AB}\left(I⊗U^{†}\right)\right)\right\}\\ = & \underset{S}{\sup}\left\{\frac{1}{\sqrt{\det\Gamma }}-\frac{1}{\sqrt{\det[\left(\Gamma +\Gamma_{U}\right)/2]}}\right\}. \end{array}$$

Since A>0, B>0 and $\frac{\Gamma +\Gamma_{U}}{2}=\left(\begin{array}{cc} A & \frac{C+CS^{T}}{2}\\ \frac{C^{T}+SC^{T}}{2} & B \end{array}\right)$, by Fischer’s inequality (p. 506, [33]), we have $\det\frac{\Gamma +\Gamma_{U}}{2}\leq \left(\det A\right)\left(\det B\right)$. Thus, we get $N\left(\rho_{AB}\right)\leq \frac{1}{\sqrt{\det\Gamma }}-\frac{1}{\sqrt{\left(\det A)(\det B\right)}}$. If $\rho_{AB}$ is a pure state, then $1=\mathrm{Tr}\left(\rho_{AB}^{2}\right)=\frac{1}{\sqrt{\det\Gamma }}$, which gives $N\left(\rho_{AB}\right)\leq 1-\frac{1}{\sqrt{\left(\det A)(\det B\right)}}.$

Notice that, by Equation (6), we have $\frac{1}{\det\Gamma }=\mathrm{Tr}{\left(\rho_{AB}^{2}\right)}^{2}\leq 1$. This implies that $N\left(\rho_{AB}\right)\leq \frac{1}{\sqrt{\det\Gamma }}-\frac{1}{\sqrt{\left(\det A)(\det B\right)}}<1$ since detA>0 and detB>0, that is, the inequality (5) is true.

To see that the upper bound 1 is sharp, consider the two-mode squeezed vacuum state $\rho \left(r\right)=S\left(r\right)|00\rangle \langle 00|S^{†}\left(r\right)$, where $S\left(r\right)=\exp\left(-r{\hat{a}}_{1}{\hat{a}}_{2}+r{\hat{a}}_{1}^{†}{\hat{a}}_{2}^{†}\right)$ is the two-mode squeezing operator with squeezed number r≥0 and |00〉 is the vacuum state ([24]). The CM of ρ(r) is $\frac{1}{2}\left(\begin{array}{cc} A_{0} & B_{0}\\ B_{0} & A_{0} \end{array}\right)$, where $A_{0}=\left(\begin{array}{cc} \exp(-2r)+\exp(2r) & 0\\ 0 & \exp(-2r)+\exp(2r) \end{array}\right)$ and $B_{0}=\left(\begin{array}{cc} -\exp(-2r)+\exp(2r) & 0\\ 0 & \exp(-2r)-\exp(2r) \end{array}\right)$. By Theorem 2, it is easily calculated that

$$N\left(\rho \left(r\right)\right)=1-\frac{8}{6+\exp(-4r)+\exp(4r)}.$$

Clearly, N(ρ(r))→1 as r→∞, thus

$$\underset{r}{\sup}N\left(\rho \left(r\right)\right)=1,$$

completeing the proof. ☐

## 4. Comparison with Other Quantum Correlations

Entanglement is one of the most important quantum correlations, being central in most quantum information protocols [1]. However, it is an extremely difficult task to verify whether a given quantum state is entangled or not. Recall that a quantum state $\rho_{AB}\in S\left(H_{A}⊗H_{B}\right)$ is said to be separable if it belongs to the closed convex hull of the set of all product states $\rho_{A}⊗\rho_{B}\in S\left(H_{A}⊗H_{B}\right)$. Note that a state $\rho_{AB}$ is separable if and only if it admits a representation $\rho_{AB}=\int_{X}\rho_{A}\left(x\right)⊗\rho_{B}\left(x\right)\pi \left(dx\right)$, where π(dx) is a Borel probability measure and $\rho_{A(B)}\left(x\right)$ is a Borel $S(H_{A(B)})$-valued function on some complete, separable metric space X [34]. One of the most useful separability criteria is the positive partial transpose (PPT) criterion, which can be found in [35,36]. The PPT criterion states that if a state is separable, then its partial transposition is positive. For discrete systems, the positivity of the partial transposition of a state is necessary and sufficient for its separability in the 2⊗2 and 2⊗3 cases. However, it is not true for higher dimensional systems [36]. For continuous systems, in [27,37], the authors extended the PPT criterion to (n+m) -mode continuous systems. It is remarkable that, for any (1+n)-mode Gaussian state, it has PPT if and only if it is separable. Furthermore, for the (1+1)-mode case, it is shown that a (1+1)-mode Gaussian state $\rho_{AB}$ is separable if and only if ${\bar{v}}_{-}\geq 1$, where ${\bar{v}}_{-}$ is the smallest symplectic eigenvalue of the CM of the partial transpose $\rho_{AB}^{T_{B}}$ [24,29].

Comparing N with the entanglement, we conjecture that there exists some positive number d<1 such that $N(\rho_{AB})\leq d$ for any (n+m)-mode separable Gaussian state $\rho_{AB}$, that is,

$$\underset{\rho_{AB}\;\mathrm{is}\mathrm{separable}}{\sup}N\left(\rho_{AB}\right)\leq d<1.$$

If this is true, then $\rho_{AB}$ is entangled when $N(\rho_{AB})>d$. This will give a criterion of entanglement for (n+m)-mode Gaussian states in terms of correlation N. Though we can not give a mathematical proof, we show that this is true for (1+1)-mode separable Gaussian states with $d\leq \frac{1}{10}$ by a numerical approach (Firstly, we randomly generated one million, five million, ten million, fifty million, one hundred million, five hundred million separable Gaussian states with a,b,|c|,|d| ranging from 1 to 2, respectively. We found that the maximum of N is smaller than 0.09. Secondly, we used the same method and extended the range to 5. Then, the maximum of N is smaller than 0.1. Thirdly, using the same method and extending the range to 10,100,1000,10000, respectively, we found that the maximum of N is still smaller than 0.1. We repeated the above computations ten times, and the result is just the same).

**Proposition** **2.** $N(\rho_{AB})\leq 0.1$ for any (1+1)-mode separable Gaussian state $\rho_{AB}$.

It is followed from Theorem 1 that the quantum correlation N exists in all entangled Gaussian states and almost all separable Gaussian states except product states. In addition, Proposition 2 can be viewed as a sufficient condition for the entanglement of two-mode Gaussian states: if $N(\rho_{AB})>0.1$, then $\rho_{AB}$ is entangled.

To have an insight into the behavior of this quantum correlation by N and to compare it with the entanglement and the discords, we consider a class of physically relevant states–squeezed thermal state (STS). This kind of Gaussian state is used by many authors to illustrate the behavior of several interesting quantum correlations [12,13]. Recall that a two-mode Gaussian state $\rho_{AB}$ is an STS if $\rho_{AB}=S\left(r\right)\nu_{1}\left({\bar{n}}_{1}\right)⊗\nu_{2}\left({\bar{n}}_{2}\right)S{\left(r\right)}^{†},$ where $\nu_{i}\left({\bar{n}}_{i}\right)=\sum_{k}\frac{{\bar{n}}_{i}^{k}}{{\left(1+{\bar{n}}_{i}\right)}^{k+1}}\left|k\rangle \langle k\right|$ is the thermal state with thermal photon number ${\bar{n}}_{i}$ (i=1,2) and $S\left(r\right)=\exp\{r\left({\hat{a}}_{1}^{†}{\hat{a}}_{2}^{†}-{\hat{a}}_{1}{\hat{a}}_{2}\right)\}$ is the two-mode squeezing operator. Particularly, when ${\bar{n}}_{1}={\bar{n}}_{2}=0$, $\rho_{AB}$ is a pure two-mode squeezed vacuum state, also known as an Einstein–Podolski–Rosen (EPR) state [24]. When ${\bar{n}}_{1}>0$ or ${\bar{n}}_{2}>0$, $\rho_{AB}$ is a mixed Gaussian state. For fixed *r*, $\rho_{AB}$ is separable (not in product form) for large enough ${\bar{n}}_{1},{\bar{n}}_{2}$. Notice that if ρ is a STS with the CM $\Gamma_{0}$ in the standard form in Equation (2), then c=−d. In this case, by Theorem 2, we have

(7) $$\begin{array}{c} N\left(\rho_{AB}\right)=\frac{1}{ab-c^{2}}-\frac{1}{ab-c^{2}/2}. \end{array}$$

Using this parametrization, one can get $a=2{\bar{n}}_{r}+1+2{\bar{n}}_{1}\left(1+{\bar{n}}_{r}\right)+2{\bar{n}}_{2}{\bar{n}}_{r}$, $b=2{\bar{n}}_{r}+1+2{\bar{n}}_{2}\left(1+{\bar{n}}_{r}\right)+2{\bar{n}}_{1}{\bar{n}}_{r}$ and $c=-d=2\left(1+{\bar{n}}_{1}+{\bar{n}}_{2}\right)\sqrt{{\bar{n}}_{r}\left(1+{\bar{n}}_{r}\right)}$, where ${\bar{n}}_{r}=\sinh^{2}r$ ([12]). Especially, if ${\bar{n}}_{1}={\bar{n}}_{2}=\bar{n}$, then $\rho_{AB}$ is called a symmetric squeezed thermal state (SSTS). Now assume that $\rho_{AB}$ is a SSTS. Then, $\rho_{AB}$ is a mixed state if and only if $\bar{n}>0$. The global purity of $\rho_{AB}$ is $\mu =\mathrm{Tr}\left(\rho_{AB}^{2}\right)=\frac{1}{{\left(1+2\bar{n}\right)}^{2}}$ and the smallest symplectic eigenvalue ${\bar{v}}_{-}$ of CM of $\rho_{AB}^{T_{B}}$ is ${\bar{v}}_{-}=\frac{1+2\bar{n}}{\exp\left(2r\right)}$. Moreover, $\rho_{AB}$ is entangled if and only if ${\bar{v}}_{-}<1$.

**Figure 1:**
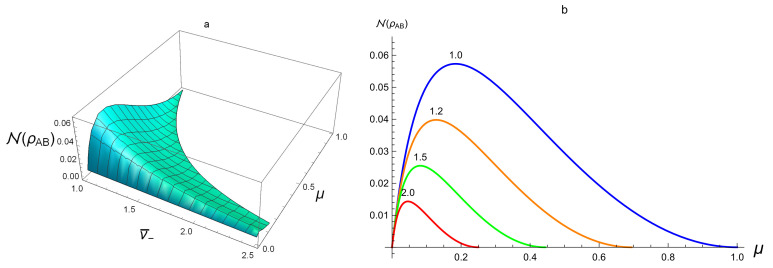
(**a**) $N(\rho_{AB})$ for separable SSTSs as a function of μ and ${\bar{v}}_{-}$; (**b**) from top to bottom, ${\bar{v}}_{-}$ = 1.0, 1.2, 1.5, 2.0.

We first discuss the relation between N and the entanglement by considering SSTS. Regard $N(\rho_{AB})$ as a function of μ and ${\bar{v}}_{-}$. From Figure 1a, for separable states, we see that the value N at the separable SSTS is always smaller than 0.06, which supports positively Proposition 2. From Figure 1b, for fixed purity μ, N turns out to be a decreasing function of ${\bar{v}}_{-}$. However, for fixed ${\bar{v}}_{-}$, N tends to 0 when μ increases.

**Figure 2:**
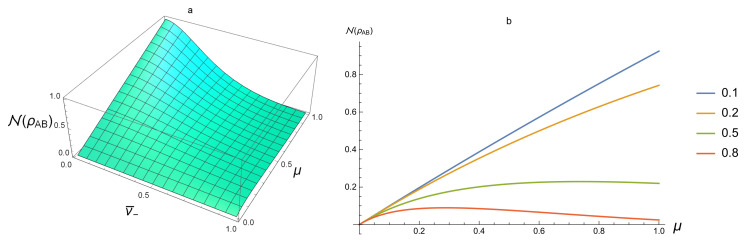
(**a**) $N(\rho_{AB})$ for entangled SSTS as a function of μ and ${\bar{v}}_{-}$; (**b**) from top to bottom, ${\bar{v}}_{-}=0.1,0.2,0.5,0.8$.

For the entangled SSTS, one sees from Figure 2a,b that the value of N is from 0 to 1. This reveals that, for some entangled SSTSs, N can be smaller than $\frac{1}{10}$. Thus, Proposition 2 is only a necessary condition for a Gaussian state to be separable. For fixed purity μ, from Figure 1b and Figure 2b, $N(\rho_{AB})$ increases when entanglement increases (that is, ${\bar{v}}_{-}\to 0$) and $\lim_{\mu \to 1,{\bar{v}}_{-}\to 0}N=1$. However, for fixed ${\bar{v}}_{-}$, the behavior of N on μ is more complex.

**Figure 3:**
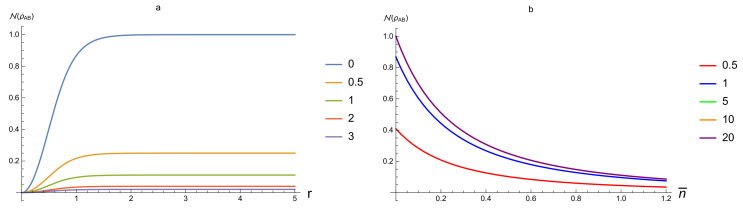
$N(\rho_{AB})$ for SSTS as a function of $\bar{n}$ and *r*. (**a**) from top to bottom $\bar{n}=0,0.5,1,2,3$; (**b**) from top to bottom r=0.5,1,5,10,20.

Regarding N as a function of *r* and $\bar{n}$, Figure 3 shows that $N(\rho_{AB})$ is an increasing function of *r* and a decreasing function of $\bar{n}$, respectively. The value of $N(\rho_{AB})$ always gains the maximum at $\bar{n}=0$, that is, at pure states. Figure 3b also shows that $N(\rho_{AB})$ almost depends only on $\bar{n}$ when *r* is large enough because the curves for r=5,10,20 are almost the same.

Recall that an *n*-mode Gaussian positive operator-valued measure (GPOVM) is a collection of positive operators Π={Π(z)} satisfying $\int_{z}\Pi \left(z\right)dz=I$, where $\Pi \left(z\right)=W\left(z\right)\omega W^{†}\left(z\right),z\in R^{2n}$ with W(z) the Weyl operators and ω an *n*-mode Gaussian state, which is called the seed of the GPOVM Π [38,39]. Let $\rho_{AB}$ be a (n+m)-mode Gaussian state and Π={Π(z)} be a GPOVM of the subsystem B. Denote by $\rho_{A}\left(z\right)=\frac{1}{p(z)}{\mathrm{Tr}}_{B}\left(\rho_{AB}I⊗\Pi \left(z\right)\right)$ the reduced state of the system A after the GPOVM Π performed on the system B, where $p\left(z\right)=\mathrm{Tr}(\rho_{AB}I⊗\Pi \left(z\right))$. Write the von Neumann entropy of a state ρ as S(ρ), that is, S(ρ)=−Tr(ρlogρ). Then, the Gaussian QD of $\rho_{AB}$ is defined as $D\left(\rho_{AB}\right)=S\left(\rho_{B}\right)-S\left(\rho_{AB}\right)+\inf_{\Pi }\int dzp\left(z\right)S\left(\rho_{A}\left(z\right)\right)$ [12,13], where the infimum takes over all GPOVMs Π performed on the system B. It is known that a (1+1)-mode Gaussian state has zero Gaussian QD if and only if it is a product state; in addition, for all separable (1+1)-mode Gaussian states, $D(\rho_{AB})\leq 1$; if the standard form of the CM of a (1+1)-mode Gaussian state $\rho_{AB}$ is as in Equation (2), then

(8) $$D\left(\rho_{AB}\right)=f\left(\sqrt{\det B_{0}}\right)+f\left(v_{-}\right)+f\left(v_{+}\right)+f\left(\sqrt{\inf_{\omega }\det E_{\omega }}\right),$$

where the infimum takes over all one-mode Gaussian states ω, $f\left(x\right)=\frac{x+1}{2}\log\frac{x+1}{2}-\frac{x-1}{2}\log\frac{x-1}{2}$, $v_{-}$ and $v_{+}$ are the symplectic eigenvalues of the CM of $\rho_{AB}$, $E_{\omega }=A_{0}-C_{0}{\left(B_{0}+\Gamma_{\omega }\right)}^{-1}C_{0}^{T}$ with $\Gamma_{\omega }$ the CM of ω. Let $\alpha =\det A_{0},\beta =\det B_{0},\gamma =\det C_{0},\delta =\det\Gamma_{0}$, then we have [13]

(9) $$\underset{\omega }{\inf}\det E_{\omega }=\left\{\begin{array}{cc} \frac{2\gamma^{2}+\left(\beta -1\right)\left(\delta -\alpha \right)+2\left|\gamma \right|\sqrt{\gamma^{2}+\left(\beta -1\right)\left(\delta -\alpha \right)}}{{\left(\beta -1\right)}^{2}} & \mathrm{if}\;{\left(\delta -\alpha \beta \right)}^{2}\leq \left(1+\beta \right)\gamma^{2}\left(\alpha +\delta \right),\\ \frac{\alpha \beta -\gamma^{2}+\delta -\sqrt{\gamma^{4}+{\left(\delta -\alpha \beta \right)}^{2}-2\gamma^{2}\left(\alpha \beta +\delta \right)}}{2\beta } & \mathrm{otherwise}. \end{array}\right.$$

In [14], the quantum GD $D_{G}$ is proposed. Consider an (n+m)-mode Gaussian state $\rho_{AB}$, its Gaussian GD is defined by $D_{G}\left(\rho_{AB}\right)=\inf_{\Pi }\left|\right|\rho_{AB}-\Pi \left(\rho_{AB}\right){\left|\right|}_{2}^{2}$, where the infimum takes over all GPOVM Π performed on system *B*, ${\left||\cdot |\right|}_{2}$ stands for the Hilbert–Schmidt norm and $\Pi \left(\rho_{AB}\right)=\int dz\left(I⊗\sqrt{\Pi (z)}\right)\rho_{AB}\left(I⊗\sqrt{\Pi (z)}\right)$. If $\rho_{AB}$ is a (1+1)-mode Gaussian state with the CM Γ as in Equation (1) and Π is an one-mode Gaussian POVM performed on mode B with seed $\omega_{B}$, then $\Pi \left(\rho_{AB}\right)=\omega_{A}⊗\omega_{B}$, where $\omega_{A}$ is a Gaussian state of which the CM $\Gamma_{\omega_{A}}=A+C{\left(B+\Gamma_{B}\right)}^{-1}C^{T}$ with $\Gamma_{\omega_{B}}$ the CM of $\omega_{B}$. It is known from [14] that

(10) $$\begin{array}{c} D_{G}\left(\rho \right)=\underset{\omega_{B}}{\inf}\left|\right|\rho_{AB}-\omega_{A}⊗\omega_{B}{\left|\right|}_{2}^{2}. \end{array}$$

Now it is clear that, for (1+1)-mode Gaussian state $\rho_{AB}$, $D_{G}\left(\rho_{AB}\right)=0$ if and only if $\rho_{AB}$ is a product state.

By Theorem 1 and the results mentioned above, $D,D_{G}$ and N describe the same quantum correlation for (1+1)-mode Gaussian states. However, from the definitions, $D,D_{G}$ use all GPOVMs, while N only employs Gaussian unitary operations, which is simpler and may consume less physical resources. Moreover, though an analytical formula of *D* is given for two-mode Gaussian states, the expression is more complex and more difficult to calculate (Equations (8) and (9)). $D_{G}$ is not handled in general and there is no analytical formula for all (1+1)-mode Gaussian states (Equation (10)). As far as we know, there are no results obtained on $D,D_{G}$ for general (n+m)-mode case.

To have a better insight into the behavior of N and $D_{G}$, we compare them in scale with the help of two-mode STS. Note that $D_{G}$ of any two-mode STS $\rho_{AB}$ is given by [14]

(11) $$\begin{array}{c} D_{G}\left(\rho_{AB}\right)=\frac{1}{ab-c^{2}}-\frac{9}{{\left(\sqrt{4ab-3c^{2}}+\sqrt{ab}\right)}^{2}}. \end{array}$$

Clearly, our formula (7) for N is simpler then formula (11) for $D_{G}$.

**Figure 4:**
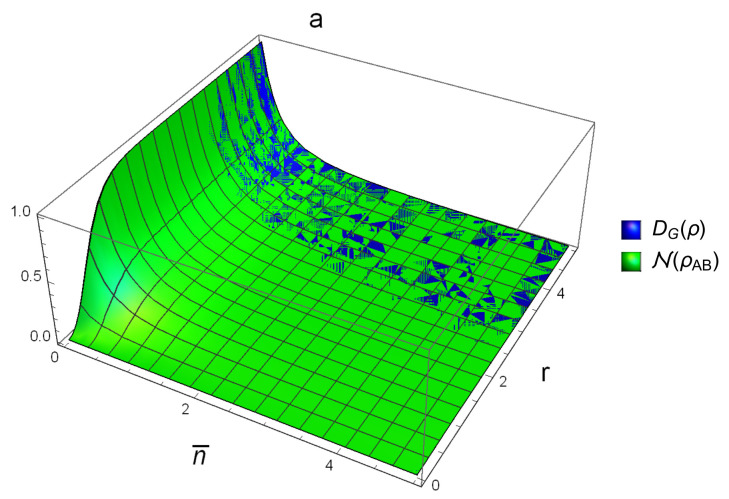
Comparison with $D_{G}\left(\rho_{AB}\right)$ for SSTS.

**Figure 5:**
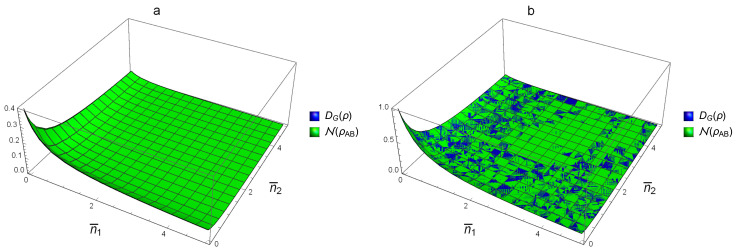
Comparison with $D_{G}\left(\rho_{AB}\right)$ for nonsymmetric STS. (**a**) and (**b**) are correspond to nonsymmetric STS with r=0.5,5, respectively.

Figure 4 and Figure 5 are plotted in terms of photo number $\bar{n}$ and squeezing parameter *r*. Figure 4 shows that, for the case of SSTS and for 0<r≤2.5, we have $D_{G}\left(\rho_{AB}\right)<N\left(\rho_{AB}\right)$. This means that N is better than $D_{G}$ when they are used to detect the correlation that they describe in the SSTS with r<2.5. Figure 5a reveals that, for the case of nonsymmetric STS and for r=0.5, we have $D_{G}\left(\rho_{AB}\right)<N\left(\rho_{AB}\right)$; that is, N is better in this situation too. However, for r=5, N and $D_{G}$ can not be compared with each other globally, which suggests that one may use $\max\{N\left(\rho_{AB}\right),D_{G}\left(\rho_{AB}\right)\}$ to detect the correlation.

## 5. Conclusions

In conclusion, we introduce a measure of quantum correlation by N for bipartite quantum states in continuous-variable systems. This measure is introduced by performing Gaussian unitary operations to a subsystem and the value of it is invariant for all quantum states under local Gaussian unitary operations. N exists in all (n+m)-mode Gaussian states except product ones. In addition, N takes values in [0,1) and the upper bound 1 is sharp. An analytical formula of N for any (1+1)-mode Gaussian states is obtained. Moreover, for any (n+m)-mode Gaussian states, an estimate of N is established in terms of its covariance matrix. Numerical evidence shows that the inequality $N(\rho_{AB})\leq 0.1$ holds for any (1+1)-mode separable Gaussian states $\rho_{AB}$, which can be viewed as a criterion of entanglement. It is worth noting that Gaussian QD, Gaussian GD and N measure the same quantum correlation for (1+1)-mode Gaussian states. However, N is easer to calculate and can be applied to any (n+m)-mode Gaussian states.
